# Dopaminergic Toxin 1-Methyl-4-Phenylpyridinium, Proteins α-Synuclein and Glia Maturation Factor Activate Mast Cells and Release Inflammatory Mediators

**DOI:** 10.1371/journal.pone.0135776

**Published:** 2015-08-14

**Authors:** Duraisamy Kempuraj, Ramasamy Thangavel, Evert Yang, Sagar Pattani, Smita Zaheer, Donna A. Santillan, Mark K. Santillan, Asgar Zaheer

**Affiliations:** 1 Veterans Affairs Health Care System, Iowa City, Iowa, United States of America; 2 Department of Neurology, Carver College of Medicine, University of Iowa Hospitals and Clinics, Iowa City, Iowa, United States of America; 3 Department of Obstetrics and Gynecology, Carver College of Medicine, University of Iowa Hospitals and Clinics, Iowa City, Iowa, United States of America; Cornell University, UNITED STATES

## Abstract

Parkinson’s disease (PD) is characterized by the presence of Lewy bodies and degeneration of dopaminergic neurons. 1-methyl-4-phenylpyridinium (MPP+), a metabolite of neurotoxin 1-methyl-4-phenyl-1,2,3,6-tetrahydropyridine (MPTP) and Lewy body component α-synuclein activates glia in PD pathogenesis. Mast cells and glia maturation factor (GMF) are implicated in neuroinflammatory conditions including Multiple Sclerosis. However, the role of mast cells in PD is not yet known. We have analyzed the effect of recombinant GMF, MPP+, α-synuclein and interleukin-33 (IL-33) on mouse bone marrow-derived cultured mast cells (BMMCs), human umbilical cord blood-derived cultured mast cells (hCBMCs) and mouse brain-derived cultured astrocytes by quantifying cytokines/chemokines released using ELISA or by detecting the expression of co-stimulatory molecules CD40 and CD40L by flow cytometry. GMF significantly released chemokine (C-C motif) ligand 2 (CCL2) from BMMCs but its release was reduced in BMMCs from GMF knockout mice. GMF, α-synuclein and MPP+ released IL-1β, β-hexosaminidase from BMMCs, and IL-8 from hCBMCs. GMF released CCL5, and IL-33- induced the expression of GMF from hCBMCs. Novel GMF expression was detected in hCBMCs and BMMCs by immunocytochemistry. GMF released tumor necrosis factor-alpha (TNF-α) from mouse astrocytes, and this release was greater in BMMC- astrocyte coculture than in individual cultures. Flow cytometry results showed increased IL-33 expression by GMF and MPP+, and GMF-induced CD40 expression in astrocytes. Proinflammatory mediator release by GMF, MPP+ and α-synuclein, as well as GMF expression by mast cells indicate a potential therapeutic target for neurodegenerative diseases including PD.

## Introduction

Mast cells are both sensors and effectors in communication between the nervous and immune systems. In the brain, mast cells reside on the brain side of the blood-brain-barrier (BBB), and interact with neurons, glia and blood vessels. Mast cells contribute to both normal cognition and emotionality functions, as well as promote deleterious brain functions [1]. Mast cells release nerve growth factor (NGF) [2] to mediate neurotransmission, neurite outgrowth and neuronal survival in the normal brain [3–5]. However, mast cells increase BBB permeability and activate astrocytes, oligodendrocytes, microglia and T cells in neuroinflammatory and neurodegenerative disease conditions [6–9]. Previous studies using mast cell deficient mice (W/Wv) showed that mast cells induce disease onset and increase disease severity in experimental autoimmune encephalomyelitis (EAE), an animal model of Multiple Sclerosis (MS) [10,11]. Mast cells are co-localized adjacent to astrocytes in the brain in neuroinflammatory conditions [3,12]. Mast cells can selectively release proinflammatory cytokines/chemokines and neuroactive mediators including interleukin-1β (IL-1β), IL-6, IL-8, IL-18, IL-33, tumor necrosis factor-alpha (TNF-α), vascular endothelial growth factor (VEGF), corticotropin-releasing hormone (CRH), granulocyte macrophage-colony stimulating factor (GM-CSF), chemokine (C-C motif) ligand 2 (CCL2) CCL5, NGF, dopamine, substance P, histamine, β-hexosaminidase, tryptase, prostaglandins, leukotrienes, reactive oxygen species (ROS), reactive nitrogen species (RNS) and nitric oxide (NO) in pathophysiological conditions [9,13–16]. Astrocytes express the receptor for mast cell histamine [17]. Protease-activated receptors (PARs) expressed on the neurons are cleaved by the mast cell proteases and mediate neuroinflammation [18]. Cross-talk between astrocytes (CD40L) and mast cells (CD40) release inflammatory molecules [3,4,19,20]. Mast cell tryptase activates rodent microglia to release TNF-α, IL-6 and ROS [21]. Mast cells form the major and important link between neurons and neuroinflammation by releasing neuroactive histamine, serotonin, peptides, kinins, leukotrienes, cytokines and chemokines, and proteolytic enzymes [22]. Mast cell granules contain dopamine and are released upon activation [23]. We have recently shown that IL-33-induced neurodegeneration in neuronal and glial cells co-culture [16].

Glia maturation factor (GMF), a neuroinflammatory mediator was isolated, sequenced and cloned by us [24–27]. GMF is expressed in astrocytes, microglia and some neurons in the mid brain including substantia nigra and other brain areas relevant to PD pathogenesis [28]. We have previously reported mechanistic and functional interactions between GMF and proinflammatory pathways in the brain cells including glial activation by GMF [16,29–31]. Communication by glial cells and mast cells contributes to the release of high levels of proinflammatory mediators in the brain. These proinflammatory factors lead to neuronal damage and cognitive impairment [19]. Microglial activation is a prominent pathological feature in rodents and primates after 1- methyl -4- phenyl -1,2,3,6-tetrahydro pyridine (MPTP) intoxication. 1-methyl-4-phenyl-pyridinium ion (MPP+), metabolite of MPTP also induces glial responses in the mice [32]. α-synuclein, a major component of Lewy bodies can activate glial cells to induce neuroinflammation [33–35]. The relationship between mast cells and GMF in PD pathogenesis is not yet known. We have investigated if GMF is expressed in mast cells and if GMF and PD-relevant stimuli (MPP+ and α-synuclein) could activate mast cells to release PD-relevant inflammatory mediators.

## Materials and Methods

### Reagents

Dulbecco’s phosphate buffered saline (DPBS), Dulbecco’s Modified Eagle Medium Nutrient Mixture F-12 (Ham) (DMEM F12), Iscove’s Modified Dulbecco’s Medium (IMDM), 2-Mercaptoethanol, GlutaMAX-1, Insulin-Transferrin-Selenium, penicillin streptomycin, fetal bovine serum were purchased from Life Technologies (Grand Island, NY). Rabbit GMF-β polyclonal antibody, Rabbit IgG control antibody and Mouse GMF-β monoclonal antibody were obtained from Proteintech (Chicago, IL). Mouse IgG1 isotype control antibody was purchased from ThermoScientific (Rockford, IL). Murine recombinant IL-3 was purchased from PeproTech (Rocky Hill, NJ). Ficoll-Paque sterile solution was obtained from GE Healthcare Bio Sciences AB (Uppsala, Sweden). Toluidine blue, 1-methyl-4-phenylpyridinium (MPP+) and p-nitrophenyl-N-acetyl-β-D-glucosaminide were from purchased Sigma (St. Louis, MO). AC133^+^ cell isolation kits were purchased from Milltenyi Biotec (Auburn, CA). Cell culture flasks and tissue culture plates were obtained from Costar (Corning Incorporated, and Corning, NY). Enzyme-linked immunosorbent assay (ELISA) kits for mouse/human IL-1β, IL-8, TNF-α, CCL2, CCL5, recombinant mouse IL-33 (rmIL-33), recombinant human IL-33 (rhIL-33), monoclonal anti-mouse IL-33-phycoerythrin conjugated Rat IgG2A, monoclonal anti-mouse CD40/TNFRSF5-Allophycocyanin conjugated Rat IgG2A, monoclonal anti-mouse CD40L/TNFSF5 Phycoerythrin conjugated Rat IgG2A antibodies and flow cytometry reagents were obtained from R&D Systems (Minneapolis, MN). ImmPRESS reagent anti-mouse Ig peroxidase, ImmPRESS reagent anti-rabbit Ig peroxidase kits, ImmPACT DAB peroxidase and avidin-biotin complex (ABC) kits were obtained from Vector Laboratories (Burlingame, CA). Recombinant α-synuclein (human) was from Enzo Life Sciences (Farmingdale, NY) and used with both human and mouse mast cells. USP (CPD) Blood pack unit cord blood collection kits were obtained from Fenwal Inc (Lake Zurich, IL) Protease inhibitor cocktail was from Roche Diagnostics (Indianapolis, IN) and phosphatase inhibitor cocktail was obtained from Cell Signaling Technology (Danvers, MA). Anti-GAPDH polyclonal antibody and mouse anti-human mast cell tryptase monoclonal antibody were purchased from Millipore (Billerica, MA).

### Mouse primary mast cell culture

We have successfully generated GMF-knockout (GMF-KO) mice in our laboratory previously, and maintain a colony of these transgenic mice for our studies [31]. Mouse (C57BL/6; Charles River, Wilmington, MA) bone marrow-derived mast cells (BMMCs) were cultured from bone marrow cells of femur from adult wild type mice (n = 40) and GMF-KO mice (n = 30) as previously described by us and others [4,10,36]. Briefly, bone marrow cells were aspirated and cultured in DMEM containing IL-3 (10 ng/ml), 10% heat-inactivated FBS, 1% Penicillin Streptomycin, 20 μM 2-mercaptoethanol, 1% L-glutamine for 4–6 weeks at 370°C in a 5% CO_2_ incubator. Non-adherent cells were depleted twice each week with the addition of complete culture medium. After 4 weeks of culture, >98% of the cells were determined to be mast cells as indicated by toluidine blue staining. Bone marrow from several mice were pooled and cultured to grow mast cells in culture. Mast cells in the culture were identified by staining with 0.1% toluidine blue as we have reported previously [36]. This study was carried out in accordance with the recommendations in the Guide for the Care and Use of Laboratory Animals of the National Institutes of Health. The protocol was approved by the Committee on the Ethics of Animal Experiments of the University of Iowa (Iowa City, IA).

### Human primary mast cell culture

Human umbilical cord blood-derived mast cells (hCBMCs) were grown by culturing human umbilical cord blood hematopoietic stem cells with stem cell factor (SCF, Millipore, Billerica, MA) and IL-6 for 12–14 weeks as we and others have previously reported [37,38]. Human umbilical cord blood (20 ml or more, n = 20) was collected in anti-coagulant citrate phosphate dextrose solution and diluted with DPBS at the Department of Obstetrics and Gynecology (University of Iowa Hospitals and Clinics, Iowa City, IA) as approved by the Institutional Review Board of the University of Iowa (IRB#200910784) [39]. Non-phagocytic mononuclear cells were separated using sterile Ficoll-Paque solution. The isolation of hematopoietic stem and progenitor cells (CD34^+^) was performed by positive selection of CD34^+^ (CD133^+^/AC133^+^) cells by magnetic-associated cell sorting (MACS) procedure using an AC133^+^ cell isolation kit as we have reported previously [37,40]. CD34^+^ cells were cultured in IMDM supplemented with 100–200 ng/ml SCF, 50 ng/ml IL-6, 10% heat-inactivated FBS, 2-mercaptoethanol and 1% penicillin-streptomycin for up to 14 weeks in tissue culture flasks at 37°C in a 5% CO_2_ incubator. During this culture period, cells were washed with DPBS every week and resuspended using fresh complete medium. The purity of hCBMCs was evaluated by immunocytochemical staining for tryptase, as we have previously reported using tryptase monoclonal antibody [37]. Human mast cells cultured over 12 weeks with >99% purity were used for the experiments. Mast cell viability was determined by Trypan blue (0.3%) exclusion method. Human umbilical cord blood is a rich source of hematopoietic progenitor cells that can develop into mast cells *in vitro* and that can be used to study the role of mast cells in various pathophysiological conditions [41]. Human mast cells and mouse mast cells differ in their inflammatory mediators (ex. proteases) as well as their responses to some stimuli such as neuropeptides. Therefore, we have evaluated both human and mouse mast cells in the present study.

### Mouse primary astrocyte culture

Pregnant C57BL/6 mice were sacrificed on the 16-17^th^ day of gestation to obtain the embryos. Primary cultures of astrocytes were developed using embryonic brains from wild type mice (n = 10) and GMF-KO mice (n = 10) as we have described previously [42,43]. Astrocytes were grown in DMEM Nutrient Mixture F-12 (Ham) (DMEM F12) with 5–10% FBS and 1% penicillin and streptomycin at 37°C in a 5% CO_2_ and 95% air atmosphere in 25 cm^2^ or 75 cm^2^ tissue culture flasks. Astrocytes grown in this method were >98% positive for the astrocyte marker glial fibrillary acidic protein (GFAP) as determined by preliminary immunocytochemistry.

### Mouse mast cell stimulation with GMF, and cytokine/chemokine assay by ELISA

BMMCs obtained from wild type mice and GMF-KO mice were plated in separate 24 well culture plates at 0.5 to 1x10^6^ cells/ml in mouse mast cell culture medium and cultured overnight at 37°C. The cells were then incubated with GMF-β (GMF-β), a custom made protein [26] for dose-response (5 to 200 ng/ml) and time-course (2, 6, 24 and 48 h) studies or IL-33 in 1% serum containing mouse mast cell culture medium. Stimulations for dose-response and time-course studies were performed such that the reactions were stopped simultaneously to avoid differences in cell conditions or releasability. After the incubation period, the culture supernatant media was collected, centrifuged and the supernatant media was stored at -80°C. CCL2 was assayed in these media by ELISA. The working concentrations of GMF or IL-33 were prepared in sterile 0.1% BSA PBS. The vehicle control treatment was carried-out with plain culture medium containing 0.1% BSA PBS in every experiment. In another set of experiments, BMMCs (0.5x10^6^ cells/ml) were incubated for dose-response and time-course effects with GMF, as well as time-course effect of MPP+ (10 μM) and α-synuclein (5 μg/ml). Supernatant media were then collected and assayed IL-1β by ELISA. In another set of experiments, human mast cells were incubated with GMF (100 ng/ml), α-synuclein (5 μg/ml) or IL-33 (50 ng/ml) in the tissue culture plates (0.5 to 1x10^6^ cells/ml) for 6, 24 or 48 h and IL-8 release was measured in the supernatant media by ELISA. We also incubated human mast cells with MPP+ (1–100 μM) for 6 h and the supernatant media was assayed for IL-8 by ELISA. In a separate experiment, we incubated hCBMCs with α-synuclein (3 μg/ml), GMF (100 ng/ml) and IL-33 (25 ng/ml) for 48 h. After incubation, supernatant media were collected and CCL5 levels were measured by ELISA using a microplate reader (Molecular Devices, Sunnyvale, CA).

### β-hexosaminidase release as an index of mast cell degranulation

As we have previously reported, β-hexosaminidase release is an index of mast cell degranulation; therefore, we assayed for this release in BMMCs [40,44]. Mast cell mediators are released immediately by degranulation followed by slow cytokine expression. BMMCs were plated (5 ×10^4^cells/well) and incubated with GMF (100 ng/ml), MPP+ (10 μM), α-synuclein (5 μg/ml) and IL-33 (50 ng/ml) for 30 min at 37°C. Control cells were treated with buffer alone as described previously. After the incubation period was completed, the supernatant fluids were collected and pellets were lysed in 1% Triton-X-100. β-hexosaminidase was assayed in the supernatants and cell lysates. Briefly, supernatants and cell lysates (50 μl) were incubated with 100 μl of reaction buffer (3.5 mg of p-nitrophenyl-N-acetyl-β-D-glucosaminide/ml of 0.04 M citrate buffer) for 90 min at 37°C and then 0.4 M glycine (50 μl) was added to stop the reaction in 96-well plate. The absorbance was then measured at 405 nm in an ELISA microplate reader. The results were expressed as % β-hexosaminidase release.

### Immunocytochemistry (ICC) for GMF in human and mouse primary mast cells

Cytospin smears of hCBMCs and BMMCs were prepared for GMF ICC. Human mast cells were first detected based on the expression of the mast cell marker tryptase. Similarly, we have confirmed the BMMCs by 0.1% toluidine blue staining as there is no similar kind of tryptase in BMMCs. We then analyzed the expression of GMF in hCBMCs and BMMCs by ICC using both the Rabbit GMF polyclonal antibody and Mouse GMF monoclonal antibody separately. These antibodies detect both mouse and human GMFs. For ICC, cytospin smears were first fixed with acetone/methanol for 5 min. GMF ICC with polyclonal antibody was carried out using ImmPRESS reagent anti-rabbit Ig peroxidase kits. The tryptase and GMF ICCs were then carried out with monoclonal antibodies using ImmPRESS reagent anti-mouse Ig peroxidase and ImmPACT DAB peroxidase substrate kit or using ABC staining kits (Rabbit IgG/Mouse IgG ABC kits) as per the kit procedures. We used Rabbit IgG Isotype control antibody and Mouse IgG1 Isotype control antibody for GMF polyclonal antibody and GMF monoclonal antibody, respectively, to confirm the specificity of primary antibody binding. Mouse IgG1 was also used as the isotype control for tryptase staining. Isotype matched control antibodies were used at the same concentrations of the respective primary antibodies. DAB peroxidase substrate produces a brown color with positive reactions indicating the presence of GMF and tryptase.

### Immunoblotting for GMF in human mast cells

Briefly, human mast cells were seeded at 1x10^6^ cells/ml in the tissue culture flasks (25 cm^2^) and stimulated with IL-33 (50 ng/ml) for 24 h at 37°C. Following the incubation period, cells were collected and lysed in RIPA cell lysis buffer (50 mM Tris-Cl pH 7.4, 150 mM NaCl, 1mM EDTA, 1% NP-40, 0.5% deoxycholate) supplemented with protease and phosphatase inhibitor cocktail and then 25 μg proteins was subjected to SDS-PAGE on 12% gels as we have reported previously [16]. The membranes were first probed for GMF using polyclonal antibody, followed by GAPDH antibody to verify equal protein loading. Densitometry of immunoblots was performed with Image J software (National Institutes of Health, Bethesda, MD). We then calculated the densitometric ratios of IL-33 treated cells and normalized to unstimulated control cells. The control untreated cell densitometric value was set as 1 and then compared with IL-33 treated cells.

### Mouse mast cells and mouse astrocyte co-culture

Glia and mast cells activate each other in neuroinflammatory conditions. Therefore, we have studied whether GMF-dependent activation of mouse mast cells and mouse astrocytes is higher in a co-culture system or in individual culture conditions. In this experiment, mouse astrocytes were first cultured in a tissue culture plate for 2 days and the mouse mast cells were seeded in the same wells containing the astrocytes at a ratio of 1:3. Astrocytes or mast cells were also cultured separately in different wells. Astrocyte medium and mast cell medium were used at 50:50 ratios in the co-culture system. These cells were incubated with GMF (100 ng/ml) for 24 h. After the incubation period was completed, the media was removed from the wells, centrifuged and supernatants were collected and stored at -80°C for TNF-α assay by ELISA.

### Detection of IL-33, CD40 or CD40L expression in mouse primary astrocytes by Flow cytometry

We have used mouse astrocytes for these experiments due to the lack of sufficient number of mast cells. Mouse astrocytes were incubated with GMF (50 ng/ml) or MPP+ (25 μM) for 72 h at 37°C in 75 cm^2^ cell culture flasks. After the incubation period was over, cells were detached by trypsinization and immediately processed for flow cytometry as per the procedure recommended by the manufacturer (R&D Systems) using monoclonal anti-mouse IL-33-phycoerythrin conjugated Rat IgG2A or monoclonal anti-mouse CD40/TNFRSF5-Allophycocyanin conjugated Rat IgG2A or monoclonal anti-mouse CD40L/TNFSF5 Phycoerythrin conjugated Rat IgG2A antibodies. The expression of IL-33, CD40 and CD40L were analyzed by flow cytometry (BD LSR II with violet, BD Biosciences, San Jose, CA) as we have reported recently [45]. Rat IgG2A Isotype control Allophycocyanin conjugated or Rat IgG2A Isotype control Phycoerythrin conjugated were used as isotype matched controls.

### Statistical analysis

Results were analyzed using GraphPad InStat 3 software. Data were presented as mean ± SEM and analyzed using One-way Analysis of Variance (ANOVA) followed by Tukey-Kramer multiple comparison tests to determine statistically significant differences between the groups. Only ANOVA and Tukey-Kramer was used unless otherwise mentioned. An unpaired t-test was used when comparing only two conditions. A p-value of <0.05 was considered statistically significant.

## Results

### GMF activates mouse primary mast cells and release CCL2

**Fig 1:**
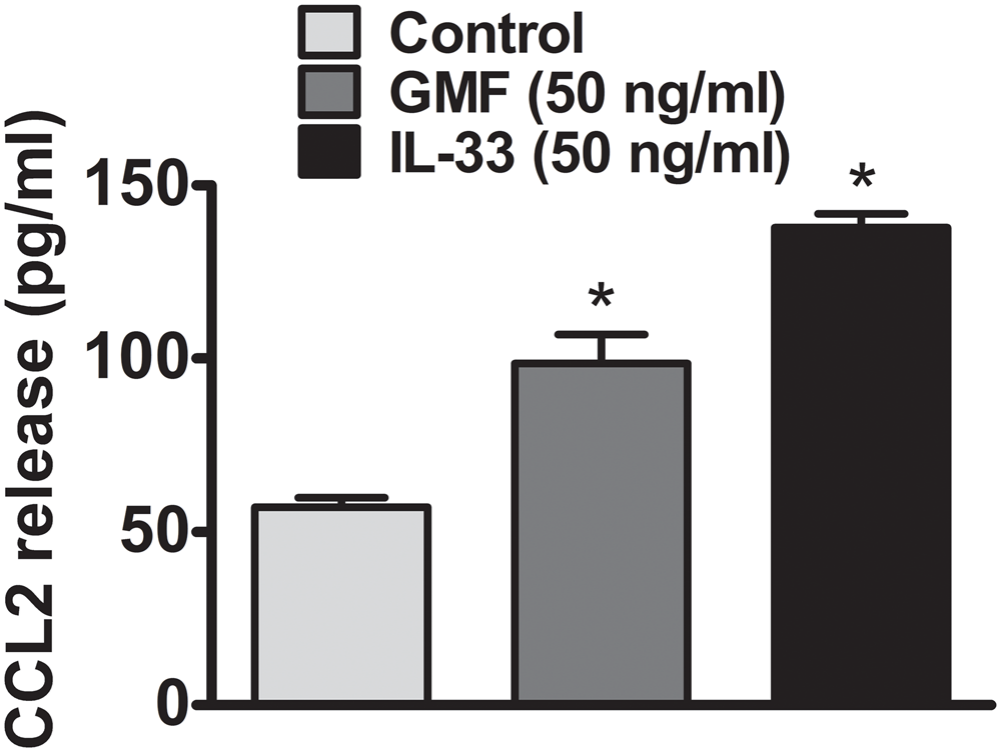
GMF activates mouse primary mast cells and release CCL2. Mouse mast cells were incubated with GMF or IL-33, both at 50 ng/ml for 48 h in vitro (n = 4). After the incubation period was over, the cell culture supernatant media was collected by centrifugation. CCL2 level was measured in the supernatant media by ELISA. IL-33 was used as a positive stimulant for the mouse mast cells at the same concentration of GMF. BMMCs incubated with either GMF or IL-33 for 48 h significantly induced CCL2 release when compared to control mast cells (*<0.05; t test, control vs treated). The control cells were treated with equal volume of medium containing 0.1% BSA PBS.

We first examined if GMF (50 ng/ml) activates BMMCs *in vitro* to release proinflammatory mediators such as chemokine CCL2. We compared the release of CCL2 induced by GMF to the release by rmIL-33 (50 ng/ml), a known positive astrocyte stimulant, at the same concentrations. BMMCs incubated with either GMF or IL-33 for 48 h showed significantly (p<0.05) increased release of CCL2 when compared to un-treated control cells (Fig 1, n = 4). IL-33 caused the release of slightly more CCL2 than by GMF.

### Decreased CCL2 release from primary mast cells obtained from GMF-KO mice

**Fig 2:**
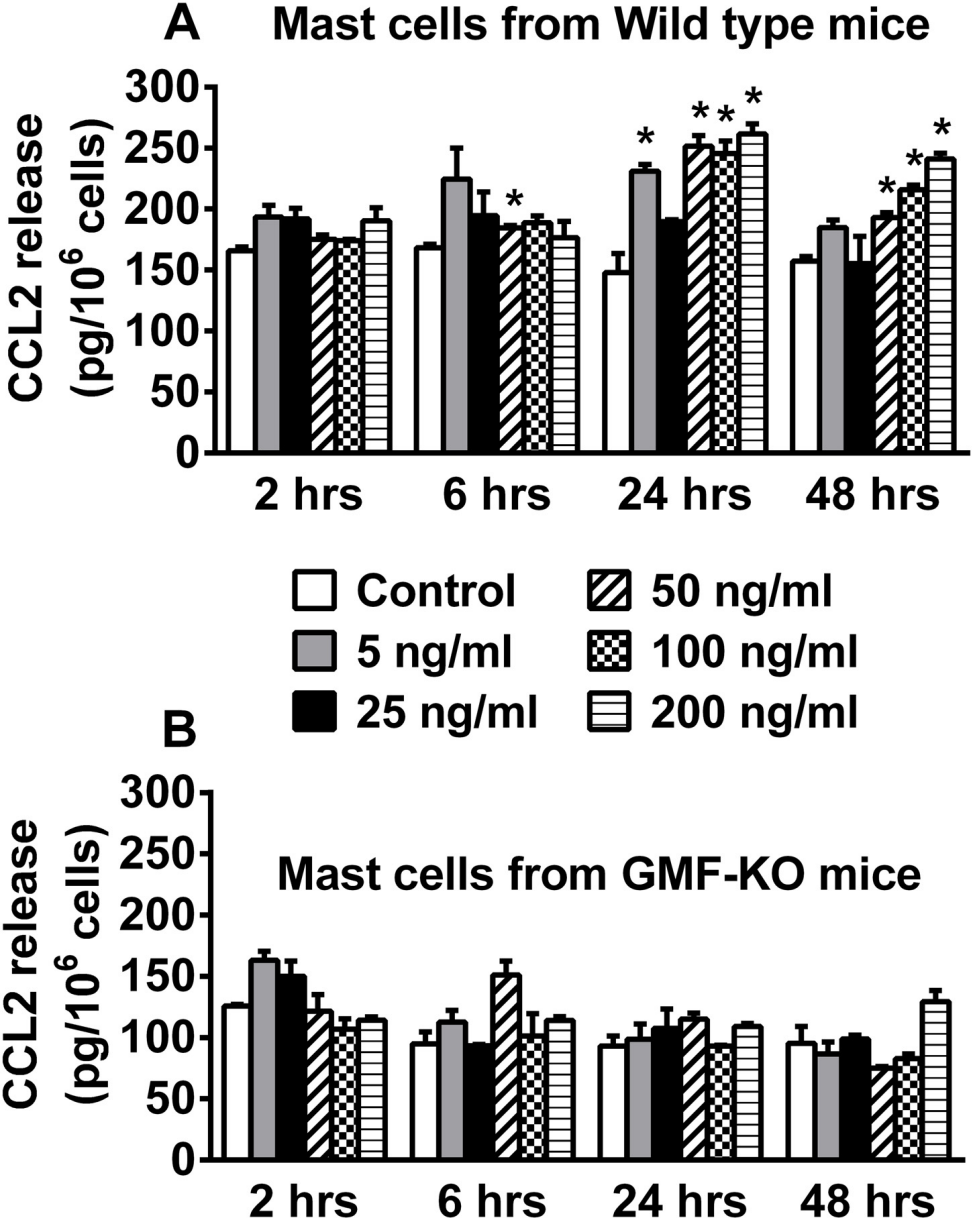
GMF induces CCL2 release from mouse primary mast cells. In dose-response and time-course studies, BMMCs were incubated with GMF and assayed CCL2 level in the culture supernatant media by ELISA (n = 4). (A) GMF significantly induced CCL2 release from BMMCs-derived from wild type mice. Further, BMMCs derived from GMF-KO mice (B) released less CCL2 when compared to CCL2 released from BBMCs (A) derived from wild type mice. The control cells were treated with equal volume of medium containing 0.1% BSA PBS. Results were presented as mean ± SEM (*p<0.05 control vs GMF treated, ANOVA and Tukey-Kramer).

Previous studies have shown decreased inflammatory mediator release from astrocytes obtained from GMF-KO mice when compared to astrocytes from wild type mice. In this study, we incubated BMMCs derived from both wild type and GMF-KO mice with GMF to determine cytokine and chemokine release based on dose-response (5, 25, 50, 100 or 200 ng/ml) and time-course (2, 6, 24 or 48 h). CCL2 released from these mast cells were measured in the supernatant media by ELISA. BMMCs derived from wild type mice showed significant (p<0.05) release of CCL2 when incubated with GMF (Fig 2A; n = 4). However, BMMCs obtained from GMF-KO mice released less of CCL2 (Fig 2B; n = 4) when compared to the level of CCL2 release from BMMCs derived from wild type mice (Fig 2A). These experiments demonstrate that lack of GMF reduces the amount of inflammatory molecules released from these mast cells.

### GMF, MPP+ and α-synuclein stimulate primary mouse mast cells to release IL-1β

**Fig 3:**
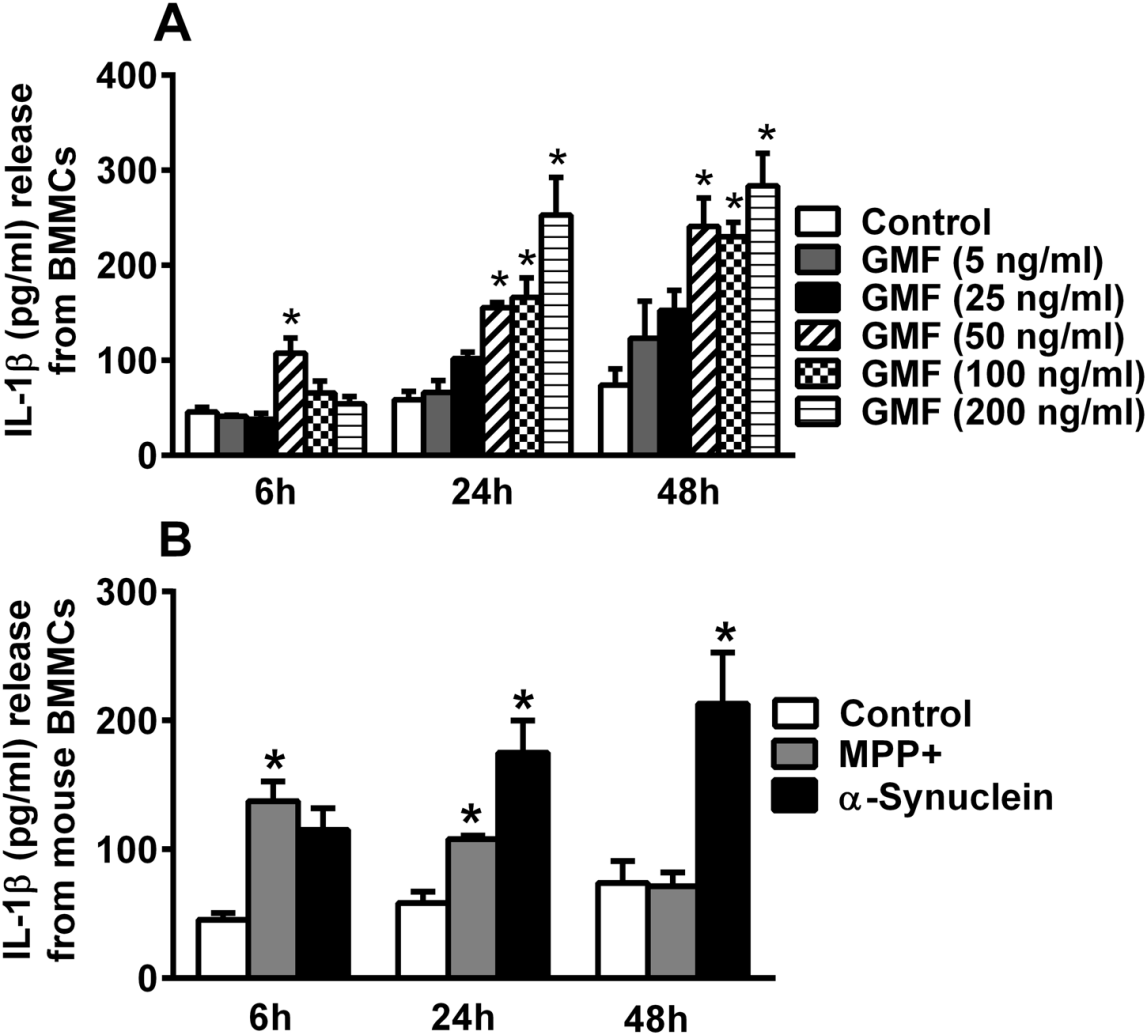
GMF, MPP+ and α-synuclein stimulates mouse mast cells to release IL-1β. Mouse mast cells were incubated with GMF for dose-response and time-course effect studies (n = 3). (A) Dose-response study showed that GMF significantly induced IL-1β release from BMMCs at 50, 100 and 200 ng/ml concentrations when compared to the release from control cells. (B) Time-course effect of MPP+ (10 μM) on BMMCs showed significant release of IL-1β at 6 h and 24 h after stimulations when compared with the release from control cells. α-synuclein (5 μg/ml) showed significant release of IL-1β after 24 h and 48 h of BMMCs stimulation when compared to the release from control cells. *p<0.05 control vs treated, ANOVA and Tukey-Kramer/Unpaired t test.

Additional time-course and dose-response effect studies on cytokine have shown that GMF significantly (p<0.05) induced IL-1β release from BMMCs at 50, 100 and 200 ng/ml concentrations when compared to the release from control cells (Fig 3A, n = 3). GMF-induced the release of IL-1β as early as 6 h from BMMCs after its stimulation. Time-course studies with MPP+ (10 μM) showed significant (p<0.05) release of IL-1β at 6 h and 24 h after stimulations when compared with the release from control cells (Fig 3B). Time-course studies with α-synuclein (5 μg/ml) showed significant (p<0.05) release of IL-1β after 24 h and 48 h of BMMCs stimulation when compared to the release from control cells (Fig 3B).

### GMF, MPP+, α-synuclein and IL-33-induced mast cell degranulation and release β-hexosaminidase

**Fig 4:**
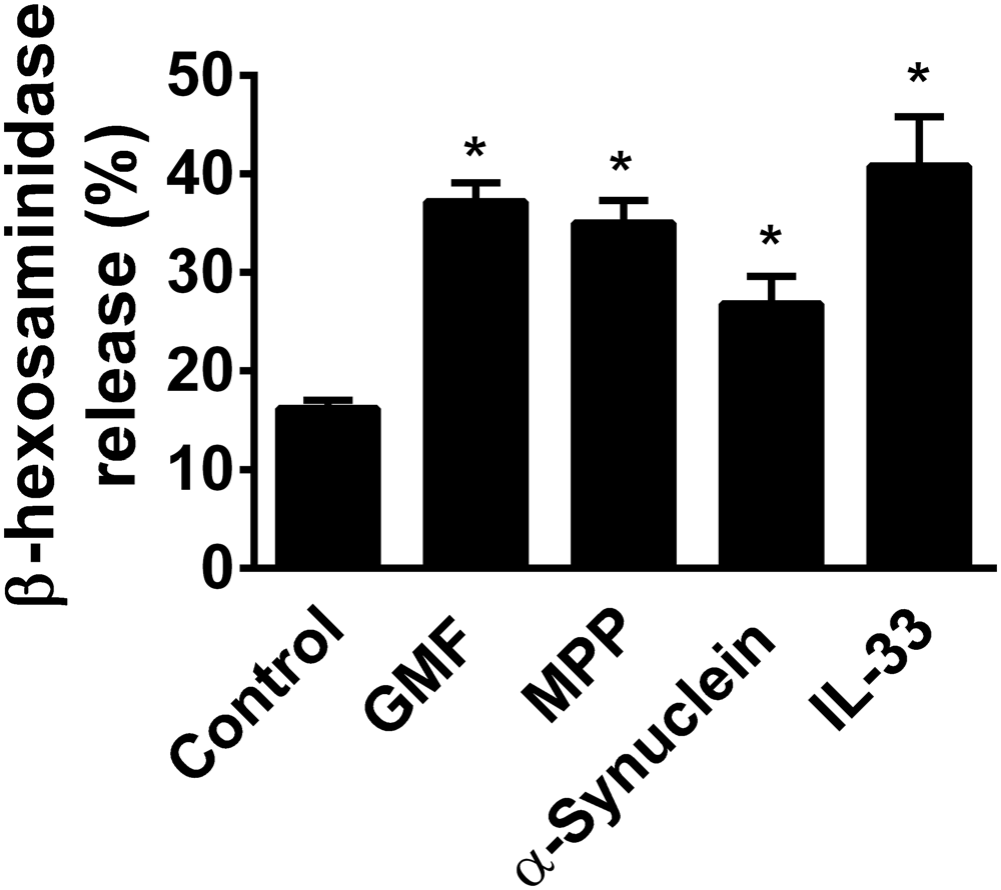
GMF, MPP+, α-synuclein and IL-33 induce the release of β-hexosaminidase as an index of mast cell degranulation. β-hexosaminidase release was assayed from BMMCs as it is as an index of mast cell degranulation (n = 5). BMMCs were (5 × 10^4^cells/well) incubated with GMF (100 ng/ml), MPP+ (10 μM), α-synuclein (5 μg/ml) or IL-33 (50 ng/ml) for 30 min at 37°C and the β-hexosaminidase release was measured in the supernatant media as well as in the cell lysates. Absorbance was measured at 405 nm in an ELISA plate reader. The results were expressed as % release. P<0.05 compared to un-treated control cells. *p<0.05 control vs treated, ANOVA and Tukey-Kramer/Unpaired t test.

BMMCs incubated with GMF (100 ng/ml), MPP+ (10 μM), α-synuclein (5 μg/ml) and IL-33 (50 ng/ml) for 30 min induced degranulation and released significantly (n = 5; p<0.05) more β-hexosaminidase when compared with the release from un-stimulated control mast cells (Fig 4). Release of β-hexosaminidase indicates activation and degranulation of mast cells associated with the release of stored granular contents.

### GMF and α-synuclein stimulate human mast cells to release chemokines IL-8 and CCL5

**Fig 5:**
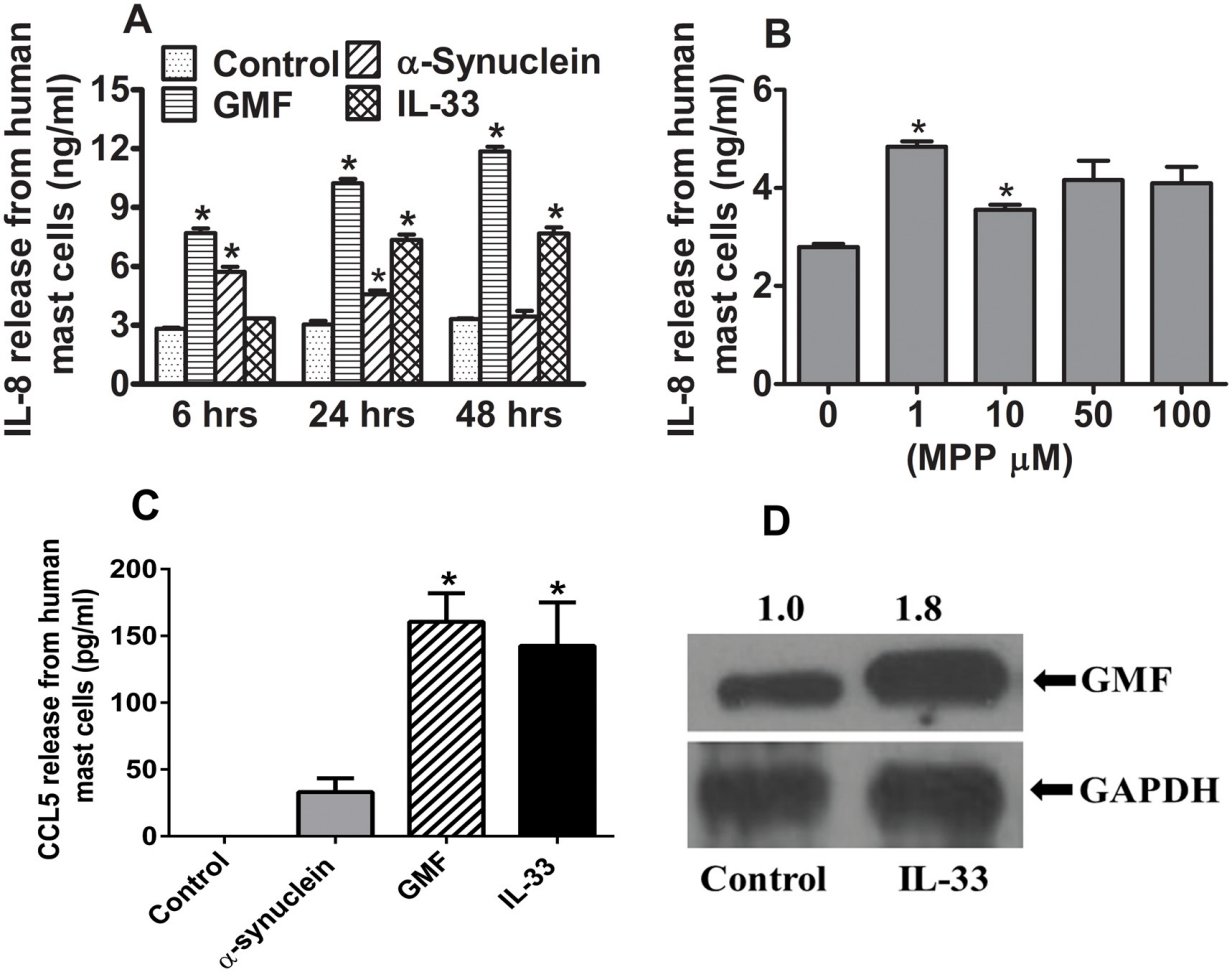
GMF stimulates human mast cells to release pro-inflammatory chemokines IL-8 and CCL5. Human mast cells were incubated with GMF (100 ng/ml), IL-33 (50 ng/ml), α-synuclein (5 μg/ml) and IL-33 for 6, 24 and 48 h, and MPP+ (1–100 μM) for 6 h for dose-response effect and the IL-8 release was measured in the supernatant media by ELISA (n = 3). (A) GMF, α-synuclein, IL-33, and (B) MPP+ significantly increased the release of IL-8 from human mast cells. We have used IL-33 as a positive control for mast cell stimulation. (C) Human mast cells were incubated with α-synuclein, GMF or IL-33 for 48 h and measured CCL5 release in the culture supernatant by ELISA. GMF and IL-33 significantly induced CCL5 release from hCMBCs when compared with control cells. *p<0.05 control vs treated, ANOVA and Tukey-Kramer/Unpaired t test. (D) Human mast cells were incubated with IL-33 (50 ng/ml) for 24 h and the expression of GMF was detected using the cell lysate by immunoblotting (n = 3). Representative results showed that human mast cells express GMF and that incubation of human mast cells with IL-33 induced GMF expression when compared to un-stimulated control cells. Densitometric analysis showed increased expression of GMF (2.1 times) after stimulation with IL-33 when compared with control unstimulated cells. GAPDH was used as loading control.

Human mast cells were incubated with GMF (100 ng/ml), α-synuclein (5 μg/ml), or IL-33 (50 ng/ml) for comparison at 6, 24 or 48 h and IL-8 release was measured in the supernatant media by ELISA (n = 3). GMF, α-synuclein and IL-33 significantly (p<0.05) increased the release of IL-8 (Fig 5A). Additionally, we have incubated human mast cells with various concentrations of MPP+ (1, 10, 50 or 100 μM) for 6 h and assayed IL-8 in the supernatant media. MPP+ at 1 and 10 μM concentrations significantly (p<0.05) released IL-8 from human mast cells when compared to untreated control cells (Fig 5B). However, at higher concentration (50 and 100 μM) MPP+ did not show enhanced IL-8 release from human mast cells. In another set of experiments, we have incubated hCBMCs with α-synuclein, GMF or IL-33 and measured CCL5 in the culture supernatant by ELISA. GMF and IL-33 significantly (p<0.05) induced CCL5 from hCBMCs when compared with control cells (Fig 5C). Although α-synuclein also induced a greater release of CCL5 when compared with control cells, the difference was not statistically significant.

### IL-33 enhances GMF expression in human mast cells

In order to study the possibility that IL-33 increases the expression of GMF, human mast cells were incubated with IL-33 (50 ng/ml) for 24 h at 37°C and the GMF level was detected by immunoblotting in mast cell lysates. Representative experimental results (n = 3; Fig 5D) showed stimulation of human mast cells with IL-33 enhanced GMF expression 1.8 fold when compared to unstimulated control cells. The mean band densitometric value of control untreated cells was set to 1 and compared to the IL-33 treated band.

### Human mast cells and mouse mast cells express GMF as determined by ICC

**Fig 6:**
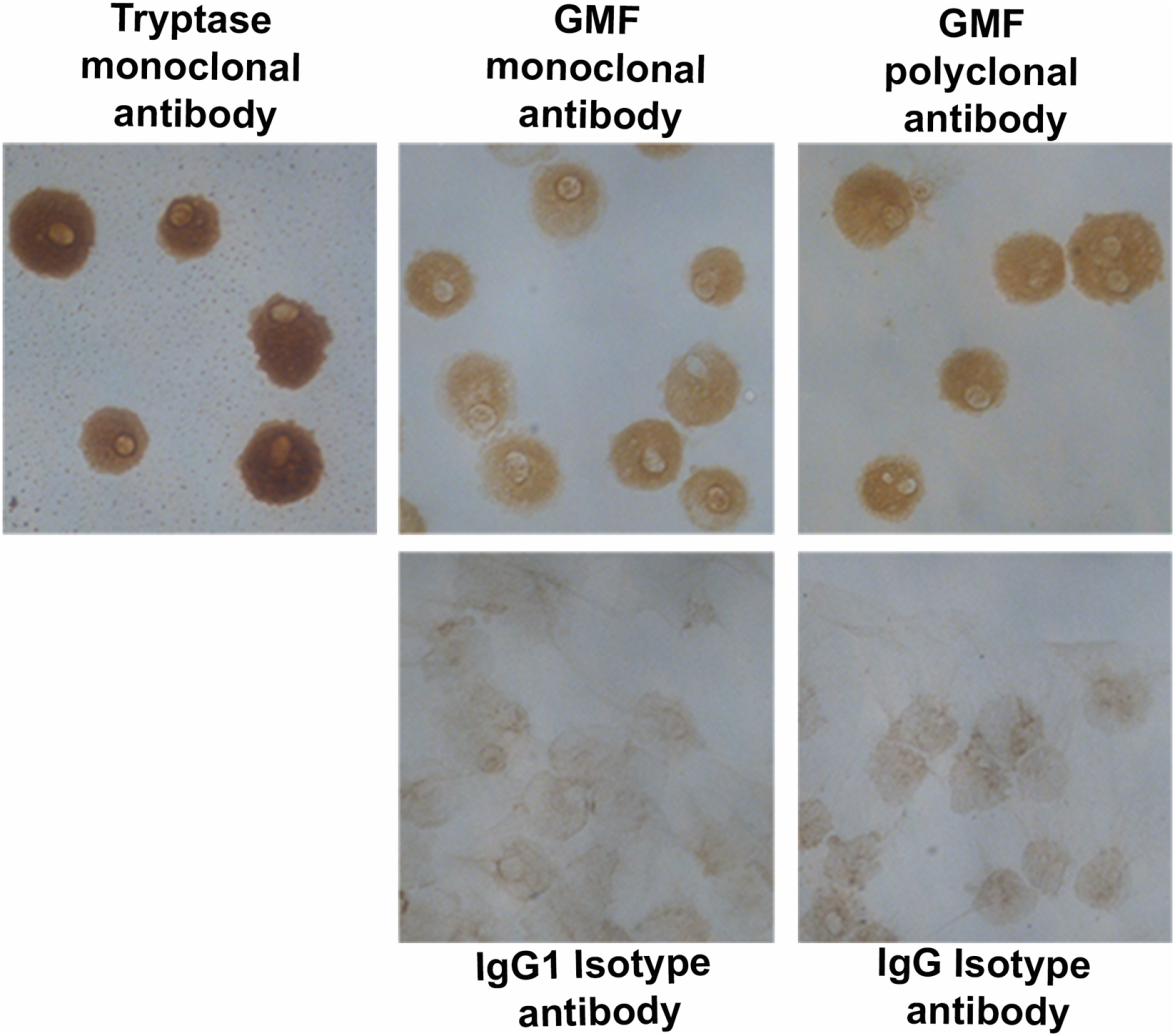
Human mast cells express GMF. Cytospin smears of cultured human mast cells were analyzed first for mast cell specific tryptase using mouse anti-human mast cell tryptase by ICC (n = 3). Mast cells were then analyzed for the expression of GMF using GMF polyclonal antibody and GMF monoclonal antibody (n = 3). GMF ICC with polyclonal antibody was carried out using ImmPRESS reagent anti-rabbit Ig peroxidase, and tryptase and another GMF ICCs were carried out with monoclonal antibodies using ImmPRESS reagent anti-mouse Ig peroxidase. ImmPACT DAB peroxidase substrate kit was used for both IHCs. Presence of brown color indicates the expression of GMF in human mast cells. Isotype matched control antibodies did not show positive reaction. Original magnification = 400x.

**Fig 7:**
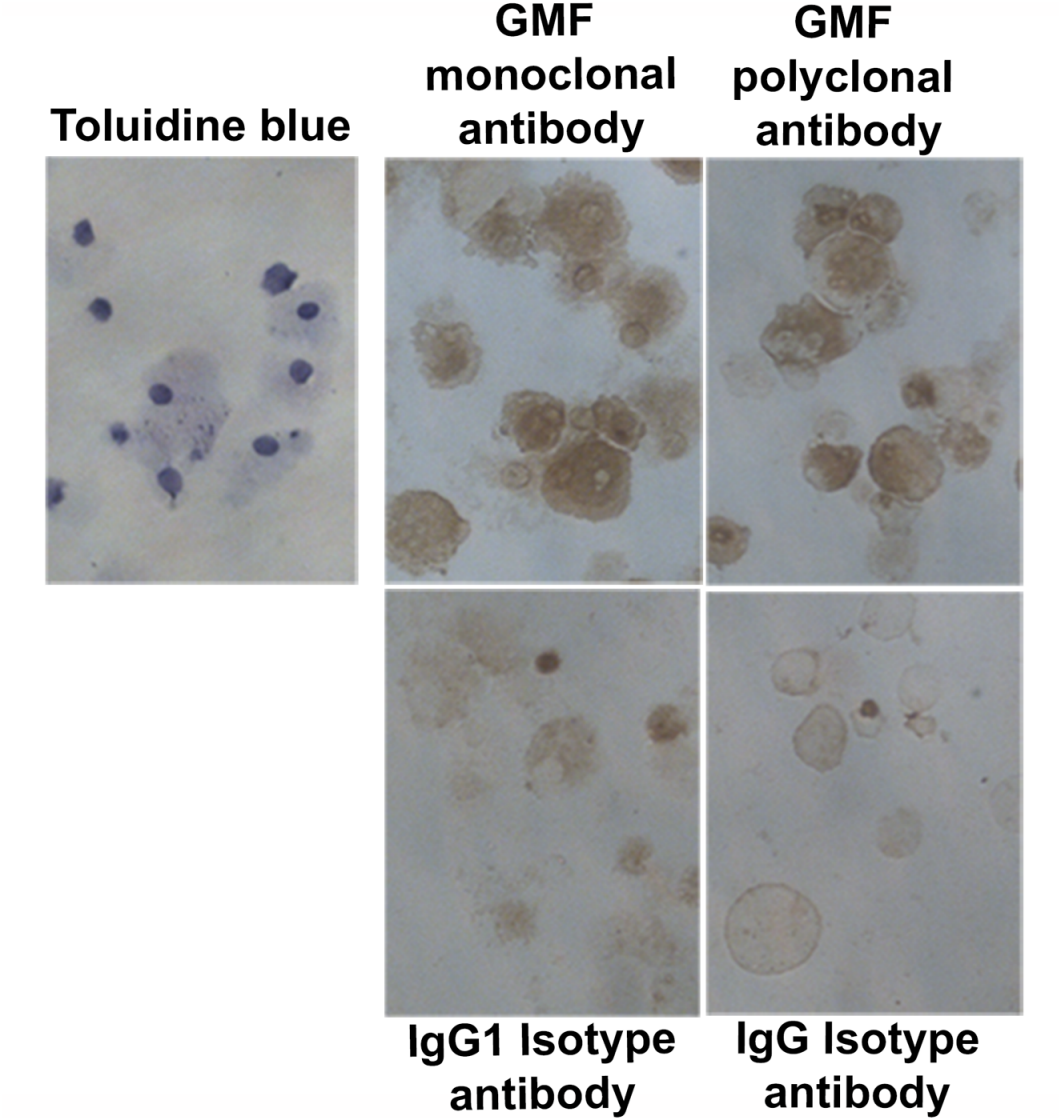
Mouse mast cells express GMF. Cytospin smears of cultured mouse mast cells were analyzed for the expression of GMF by ICC using both polyclonal antibody and monoclonal antibodies. Mouse mast cells were positive for GMF (brown color; n = 3). Isotype matched control antibodies did not show positive reactions. Mouse mast cells were identified by 0.1% toluidine blue staining. Original magnification = 400x.

We have analyzed the expression of neuropeptide GMF, in human mast cells and mouse mast cells by ICC using both GMF polyclonal and monoclonal antibodies for confirmation (Fig 6). In this study, human mast cells were first confirmed by the presence of mast cell specific tryptase (Fig 6 brown color) by ICC. Human mast cells were then analyzed for the expression of GMF by both polyclonal and monoclonal antibodies (Fig 6). We demonstrated that human mast cells were positive for GMF (brown color; n = 3). Isotype matched control antibodies did not show positive staining. Similarly, we then analyzed mouse mast cells for the expression of GMF by using both polyclonal and monoclonal antibodies (Fig 7). We demonstrated that mouse mast cells were also positive for GMF (brown color; n = 3). Again, isotype matched control antibodies did not show positive staining. Mouse mast cells were identified by 0.1% toluidine blue staining (Fig 7).

### Mouse mast cell and mouse astrocyte co-culture enhances TNF-α release

**Fig 8:**
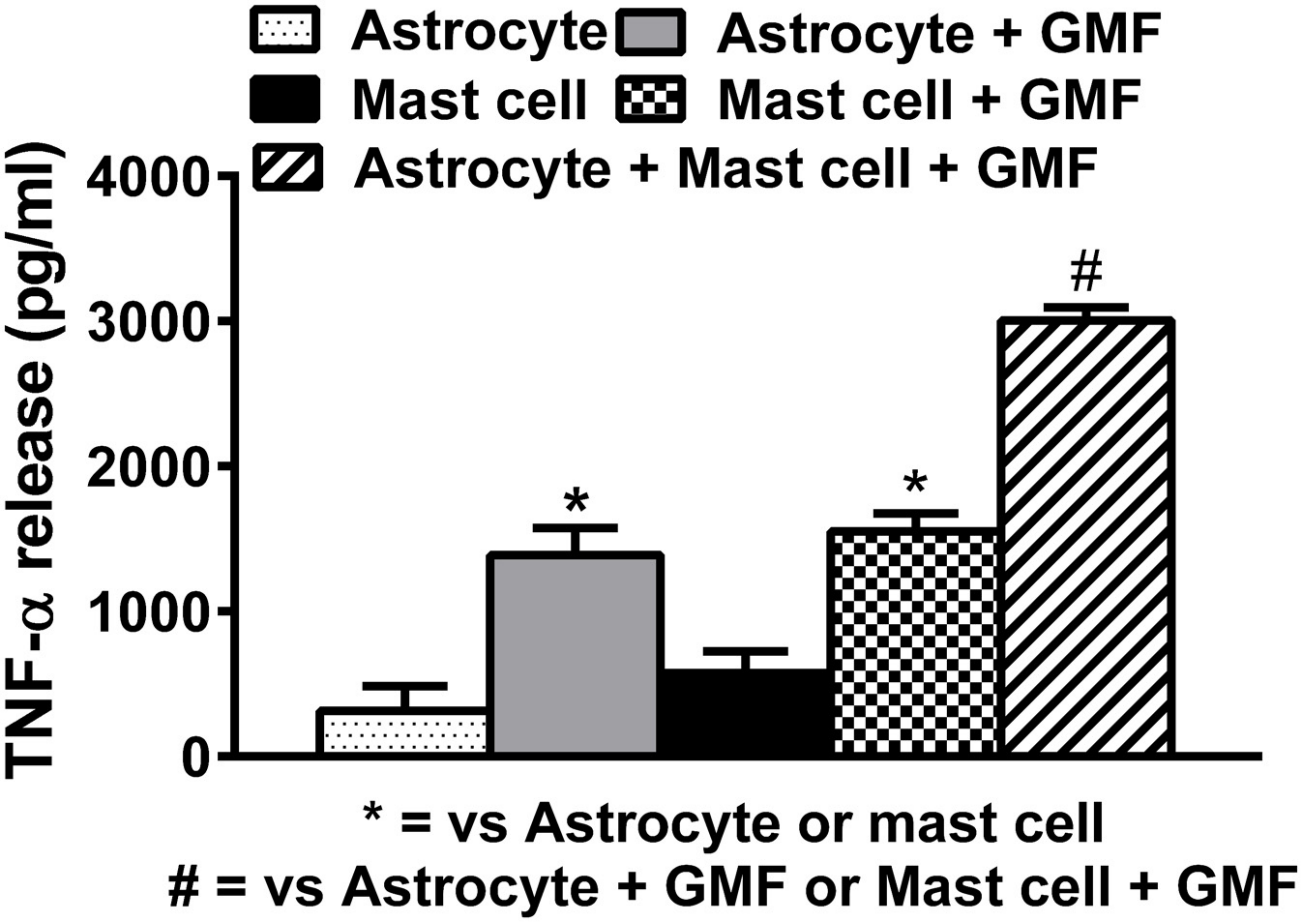
Mouse mast cells and mouse astrocyte co-culture. Mouse mast cells and mouse astrocytes were co-cultured in tissue culture plate to evaluate the effect of GMF (100 ng/ml) stimulation individually on either mast cells or astrocytes or in a co-culture system consisting of both mast cells and astrocytes in the same well (n = 4). Cells were incubated with GMF for 24 h and the supernatant media were collected by centrifugation. TNF-α level was measured by ELISA. More TNF-α release was observed from astrocyte and mast cell co-culture system than released either from astrocyte alone or mast cells alone upon incubation with GMF. # = compared to astrocyte + GMF or mast cells + GMF. *p<0.05, = compared to respective unstimulated control cells, ANOVA and Tukey-Kramer.

We cultured mouse mast cells and mouse astrocytes to evaluate the effect of GMF (100 ng/ml) stimulation individually on either mast cells or astrocytes or in an astrocyte and mast cell co-culture system (n = 4). Our results showed increased TNF-α release from astrocyte and mast cell co-culture system than released either from astrocytes alone or mast cells alone upon incubation with GMF (Fig 8) for 24 h. # represents significantly (p<0.05) increased TNF-α release when compared to either by mast cells + GMF or astrocyte + GMF conditions.

### GMF and MPP+ induce IL-33 or CD40 expression in mouse astrocytes as determined by flow cytometry

**Fig 9:**
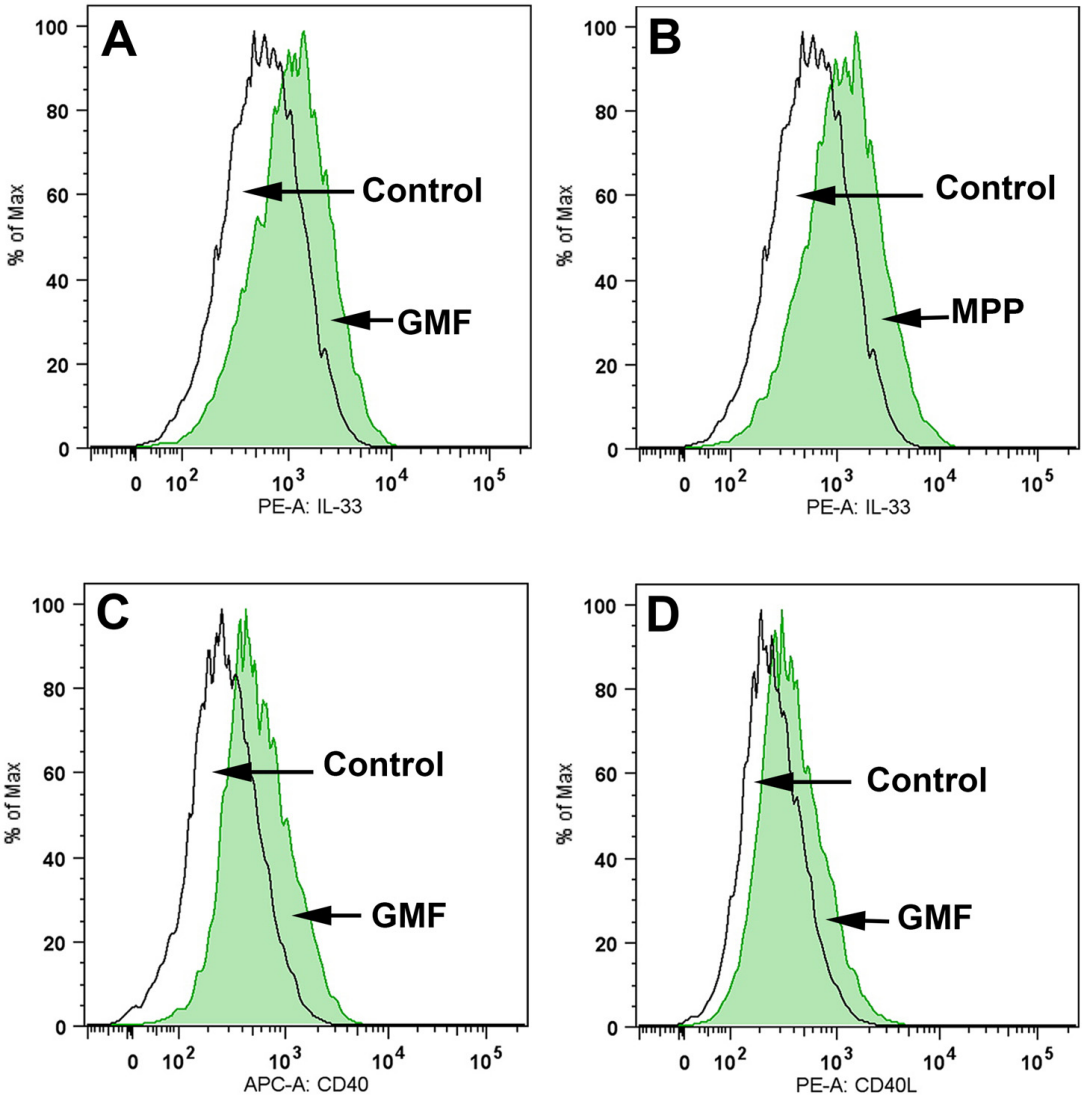
GMF and MPP+ induce IL-33 or CD40 expression in mouse astrocytes as determined by flow cytometry. Astrocytes were incubated with GMF (50 ng/ml) or MPP+ (25 μM) for 72 h at 37°C in vitro and the expression of IL-33, CD40 or CD40L were analyzed by flow cytometry using monoclonal anti-mouse IL-33-phycoerythrin or monoclonal anti-mouse CD40/TNFRSF5-APC (n = 3). GMF and MPP+ increased IL-33 expression (A, B; green color) when compared to un-treated control cells. Incubation of mouse astrocytes with GMF also induced the expression of (C) CD40, and to some extent (D) CD40L.

Several previous studies have shown that CD40 and CD40L interaction activate cells to release inflammatory mediators. Here, we showed that GMF and MPP+ induced IL-33 expression in mouse astrocytes (Fig 9A and 9B; n = 3). Astrocytes were incubated with GMF (50 ng/ml) or MPP+ (25 μM) for 72 h at 37°C and the expression of IL-33, CD40 or CD40L were analyzed by flow cytometry (BD LSR II with violet) using monoclonal anti-mouse IL-33-phycoerythrin or monoclonal anti-mouse CD40/TNFRSF5-APC or anti-mCD40L/TNFSF5 antibodies. We found that GMF upregulated the expression of IL-33 (Fig 9A; green color), CD40 (Fig 9C; green color) and CD40L (Fig 9D; green color) when compared to untreated control cells. MPP+ increased IL-33 expression in the astrocytes (Fig 9B; green color).

## Discussion

The involvement of mast cells in neuroinflammation is already well established [46]. The present study demonstrates that PD-relevant stimulants such as MPP+, α-synuclein and GMF activate mast cells to release neuroinflammatory mediators known to involve neurodegeneration. We demonstrate that MPP+, α-synuclein or GMF activate mast cells to release neurotoxic cytokines IL-1β and TNF-α as well as chemokines IL-8, CCL2 and CCL5, as well as granule stored β-hexosaminidase. Our results show that the GMF knockout condition reduces the activation and release of chemokine CCL2 from mouse mast cells. We also demonstrate novel GMF expression in human and mouse mast cells and that IL-33-induced the expression of GMF in human mast cells. We also found that GMF and MPP+ up-regulate the expression of danger signal cytokine IL-33, and GMF induces the expression of co-stimulatory molecule CD40 in the mouse astrocytes.

Mast cells play an important role in neuroinflammation and are co-localized adjacent to astrocytes in neuroinflammatory conditions [3,12]. Interestingly, studies have shown that bone marrow-derived mast cells or peripheral mast cells infiltrate the brain in pathophysiological conditions [47–49]. Peripheral mast cells can also influence the CNS [49]. Mast cells are resident in the CNS [50] and are able to cross BBB from the periphery into the brain in neuroinflammation [19]. Mast cells recruit and activate other inflammatory cells and induce vasodilation during neuroinflammation [51]. Mast cells can selectively release several cytokines/chemokines and neuroactive mediators including ROS, RNS and NO depending upon the tissue microenvironment [9,13–16,51]. These findings have shown that mast cells could influence neuroinflammation leading to neurodegeneration. Our present study has shown the release of IL-1β, IL-8, IL-33, CCL2 and CCL5 from mast cells when incubated with PD-relevant stimuli. These cytokines/chemokines are implicated in the pathogenesis of PD. Brain mast cells and glial cells interact and activate each other during neuroinflammatory responses. In fact, microglia and mast cells are suggested as two tracks leading to neuroinflammation. Additionally, mast cells are both a target and source of several neuropeptides that can mediate neuroinflammation [19,22]. Previous studies have shown that mast cells cross-talk with astrocytes, oligodendrocytes and microglia in chronic neurodegenerative diseases [19,50,52]. Glial cells, neurons and mast cells communicate through CD40L, CD40, toll-like receptor 2 (TLR2), TLR4, protease-activated receptor 2 (PAR2), CXCR4/CXCL12 and C5aR to promote glial cells migration and activation, associated with neurotoxic mediator release in neuroinflammation [4,19,21,53]. Mast cells acts as a major link between neurons and neuroinflammation [22]. Glycolytic enzyme β-hexosaminidase release is used as a marker of mast cell degranulation [54]. Our results from the present study on mast cells and GMF further strengthen this link between mast cells and glial cells in mediating neuroinflammation/neuronal death.

GMF is a prominent mediator of neuroinflammation leading to the death of neurons in the CNS [55]. We have previously shown up-regulation of GMF in the CNS of neurodegenerative diseases [56,57]. In the present study, we have investigated the expression of GMF in human and mouse mast cells, as GMF was also reported in several extra CNS cells. Our results demonstrate that mouse mast cells as well as human mast cells express GMF. Mast cells could release stored or newly synthesized GMF during neuroinflammatory conditions along with other proinflammatory mediators, probably in response to MPP+, α-synuclein or other PD-relevant proinflammatory molecules in PD. This could be possible as our present study has shown the activation of mast cells by MPP+ and α-synuclein and release of proinflammatory mediators. GMF may act in an autocrine and paracrine manner in the activation/degranulation of mast cells in the CNS. Increased levels of plasma α-synuclein have been previously reported in PD patients [58] and this may activate brain mast cells to release inflammatory mediators. Additionally, α-synuclein [59], GMF [16,55] and MPP+ [60] are known to activate glial cells and induce neuroinflammation [33–35]. Similar to these findings in glia, we also found that α-synuclein, GMF [16,55] and MPP+ activates human or mouse mast cells to release IL-8, CCL2, CCL5, TNF-α or IL-1β *in vitro*. In the present study we analyzed both TNF-α and IL-1β because both are known neurotoxic cytokines which cause neuronal damage and neuronal death [61]. Elevated levels of proinflammatory cytokines such as IL-1β and TNF-α lead to increased production of inducible nitric oxide synthase (iNOS), secretion of nitric oxide (NO), oxidative stress, neuronal stress, neuronal dysfunction and neuronal death in the brains of human PD as well as animal models of PD [62]. Our demonstration that GMF-induced significant release of TNF-α as well as IL-1β from mast cells in the present study indicates that mast cell activation could mediate neuronal degeneration in PD pathogenesis. Chemokines IL-8, CCL2 and CCL5 released from mast cells in response to PD-relevant stimulant could increase the infiltration of other inflammatory cells into the brain in PD. α-synuclein is also implicated in glial activation, oxidative stress, neuronal dysfunction, neuroinflammation and neurodegeneration by activating microglia [59] and therefore we have investigated its effect on mast cells in the present study. α-synuclein can cause dysfunction of mitochondria especially in the dopaminergic neurons in the nigrostriatal pathways in PD. As α-synuclein activates glial cells, it could also activate adjacent mast cells in neuroinflammatory conditions. It has been reported that α-synuclein overexpression, increased intracellular levels and toxic oligomer formation induced microglial activation, neuronal dysfunction and neuronal death. Several mechanistic studies have shown that α-synuclein misfolding affects mitochondria, proteasome and lysosome functions leading to α-synuclein—induced oxidative stress [63,64]. Our results that α-synuclein activates mast cells to release cytokines, chemokines and β-hexosaminidase indicates α-synuclein can activate mast cells and induce neuroinflammation/neurodegeneration in PD. In fact, increased α-synuclein increase the MPP+ mediated mitochondrial dysfunctions in PD [65]. MPP+ from neurons or glial cells could activate mast cells in the brain during the neurodegeneration process. Previous studies show that monomeric as well as aggregated α-synuclein can activate microglia [66,67] and the same can activate mast cells also. We have previously reported reduced expression of inflammatory cytokines in astrocytes and microglial cells obtained from GMF-KO mice than from wild type mice, and return of increased levels in GMF-KO cells reconstituted to overexpress GMF with an adenoviral construct [29]. The above study has demonstrated that β-amyloid-induced-production of proinflammatory cytokines/chemokines were reduced in GMF-KO mice brain and brain cells *in vitro* [29]. However, our future studies will focus on whether the synthesis or release of cytokines/chemokines is affected in GMF-KO mast cells.

GMF is known to activate astrocytes through p38 MAPK and NF-kB signaling pathways [42,55]; inhibition of MAPKs-mediated NF-kB activation pathways in GMF-KO glial cells reduced the cytokine or chemokine release *in vitro* [68]. Our present study also showed that BMMCs obtained from GMF-KO mice released less CCL2 when compared to CCL2 released from BMMCs obtained from wild type mice, demonstrating that the lack of GMF could reduce the amount of inflammatory mediator release from mast cells similar to glial cells in the brain. This is consistent with our previous findings in GMF-KO condition. The reduced release of CCL2 from GMF-KO mast cells observed in the present study could be due to the inhibition of MAPKs and NF-kB activation. Similarly, we have recently demonstrated that GMF-deficiency in astrocytes upregulates the antioxidant status and limit the extent of lipid peroxidation and production of ROS along with diminished NF-κB-mediated inflammatory responses in MPP^+^-induced toxicity [68].

We have recently reported that GMF-induced IL-33 release, and that IL-33 augments GMF-mediated TNF-α release from mouse astrocytes [16]. Furthermore, IL-33 induced CCL2, TNF-α and nitric oxide release through the phosphorylation of ERK and induced neurodegeneration in mouse astrocytes *in vitro* [16]. Our studies have also shown that IL-33 is upregulated in the glia of neurodegenerative diseases such as in Alzheimer’s disease brain [45], and MPP+ induced IL-33 release from mouse astrocytes *in vitro* [68]. Our present study further show IL-33 upregulates the expression of GMF in human mast cells indicating the increased expression of GMF when mast cells are activated in inflammatory conditions by IL-33. In the brain, IL-33 from glia may act on mast cells to release GMF which in turn may act on glial cells to release inflammatory mediators and vice versa in neuroinflammatory conditions. Moreover, mast cells act as sensors of cell injury and necrosis through IL-33 as reported previously [69] and therefore mast cells could detect neuronal damage and neurodegeneration and exacerbate inflammation through releasing additional GMF in neuroinflammatory conditions. Several previous studies have shown that IL-33 is a potent activator of mast cells to release various inflammatory molecules *in vitro* and that mast cells also synthesize and secrete IL-33 [70,71]. It is known that IL-33 produced by astrocytes induce microglial proliferation and activation, to release proinflammatory cytokines and NO in the CNS [72]. Therefore, we have used IL-33 as a positive stimulant for mast cells to compare the extent of stimulation by GMF, MPP+ or α-synuclein in the present study. We have analyzed chemokines CCL2, CCL5 and IL-8; as well as cytokines TNF-α and IL-1β as they are known to be involved in the pathogenesis of neurodegenerative diseases including PD. We analyzed IL-8 because it is released only from human mast cells but not from mouse mast cells and thus it is an indicator of human mast cell activation. We have assayed mast cell granule stored β-hexosaminidase to know if PD-relevant stimulants induce degranulation of mast cells. Unlike slow cytokine release, mast cell degranulation, release histamine or β-hexosaminidase within 10 to 15 min of stimulation. Furthermore, results from our present study showed that GMF-induces significantly more TNF-α release from astrocytes and mast cells co-culture system than released from astrocytes or mast cells cultured individually.

CD40 and CD40L interaction activate inflammatory cells to release inflammatory mediators. In this study, we have shown that GMF and MPP+ upregulate the expression of IL-33 and that GMF also increased CD40 expression in astrocytes, indicating GMF could exacerbates proinflammatory pathways through glial cells. Glial expression of GMF at the cell surface [73] could activate the adjacent mast cells in the brain. Previous study has shown that increased loss of dopaminergic neuron was associated with an increased microglial and astroglial activation associated with increased production of proinflammatory mediators [74]. Glial cells as well as brain mast cells can function as a double-edged sword, with both neurotoxic and neurotrophic effects. Further studies are required to analyze mast cell activation in the PD brains, expression of GMF at mRNA level, GMF storage in the mast cells, as well as the mechanism of mast cell activation to release GMF. In conclusion, our present preliminary study suggest that activation of mast cells by GMF, MPP+ and α-synuclein and release of proinflammatory and neurotoxic mediators along with the expression of GMF by mast cells indicate new therapeutic target for neurodegenerative diseases including Parkinson’s disease.
